# Supplementary material for: Building wet planets through high-pressure magma–hydrogen reactions
Source: Nature. 2025 Oct 29;646(8087):1069–74. doi: 10.1038/s41586-025-09630-7 (PMC12571897; doi:10.1038/s41586-025-09630-7)
Supplement: Supplementary file 1 — This file contains Supplementary Discussion sections 1–14. [file 41586_2025_9630_MOESM1_ESM.pdf]

nature portfolio

https://doi.org/10.1038/s41586-025-09630-7

Supplementary information

Building wet planets through high-pressure magma–hydrogen reactions

In the format provided by the authors and unedited

Supplementary Information

1 Diamond embrittlement by hydrogen in laser-heated diamond-anvil cell experiments

In high-P experiments, it has been extremely challenging to melt both hydrogen and silicates together (>2000 K). As the smallest atom, hydrogen can diffuse into the crystal structures of experimental assembly materials (including diamond), causing embrittlement and ultimately leading to experimental failure[75]. Such embrittlement of anvil and gasket materials makes it impossible to investigate essential chemical interactions to understand volatile-rich planets.

2 SCO-1, 2, 3, 4, and 5

$Fe_{1-y}Si_y$ All Fe-Si alloy that was observed was in the B2 structure and the B20 structure was not observed in this pressure range, which is different from the behaviors observed in H-free Fe-Si alloy[76]. A recent study found that the B2 structure can be further stabilized over B20 by hydrogen[77]. In run SCO-3, the unit-cell volume of the B2 phase is 22.30 Å^3 at 8 GPa, ∼8.5% larger than the expected volume from the equation of state of B2 FeSi with Si/Fe = 1 (or $y = 0.5$) (ref.[76]). Similar trends were also observed in other SCO runs in this pressure range. This observation could either be due to hydrogen incorporation or a lower concentration of Si (ref.[78]). This expansion persists through decompression to 1 bar suggesting that the expansion is likely not due to hydrogen incorporation in Fe metal, as it is known to dehydrogenate below 3.5 GPa (ref.[79]). This is in agreement with ref.[77] who found that B2 FeSi in a pure H medium does not take on a significant amount of H like Fe metal does. The atomic volumes (V/Z, volume per atom in the unit cell) at 8 GPa and 1 bar are both in close agreement with that of the DO_3 phase of $Fe_{0.73}Si_{0.27}$ (ref.[76]), indicating this phase has a composition similar to this phase. At 1 bar after decompression, we still observed B2 Fe-Si alloy alongside dehydrogenated bcc Fe metal.

FeH_x At this pressure range, FeH_x phases were observed in all the runs but SCO-2. Both the fcc and dhcp structures were observed. In run SCO-3 at 8 GPa, the observed unit-cell volume of fcc FeH_x, 45.91 Å^3, was higher than the expected value for Fe metal without H, 42.94 Å^3, but smaller than that of FeH, 56.42 Å^3 (ref.[80]). In order to calculate the content of hydrogen in FeH_x, we used the method developed in ref.[74] whereby the volume expansion by hydrogen incorporation was calculated at different pressures using the equations of state of FeH_x from density functional theory calculations. The method yields $FeH_{0.22}$ for the observed volume (Extended Data Fig. 7). In the same run, the dhcp structured FeH_x has a unit-cell volume of 46.32 Å^3 compared to the expected unit-cell volume of dhcp FeH_x with $x \approx 1$ of ∼52.5 Å^3 (ref.[81]). However, its volume is expanded by 9.6% compared to an estimate for hypothetical H-free Fe dhcp structure[82], again indicating substoichiometric alloying ($x \approx 0.4$). Upon decompression, FeH_x alloys are known to convert to H-free bcc Fe (ref.[79]) and we observed the same. The bcc iron shows a small unit-cell parameter of 2.843±0.003 Å compared to the expected value for pure bcc Fe metal, 2.867 Å (ref.[83]). The smaller unit-cell parameter can be explained by a small amount of Si in the quenched bcc phase.

MgO MgO was observed in all the SCO runs in this pressure range. Upon decompression to 1 bar, the unit-cell volume of MgO was 74.66±0.07 Å^3 in run SCO-3, in line with the known value of 74.71 Å^3 of pure MgO (ref.[84]), indicating no Fe^{2+} is present in the structure after heating, consistent with reduction of all Fe^{2+} in the starting materials. The result is also consistent with a recent experiment on (Mg,Fe)O in a hydrogen medium[23].

3 SCO-6, 7, 8, and 9

$Fe_{1-y}Si_y$ We observed Fe_5Si_3 (or $y = 0.38$) in all the runs in this pressure range. B2 FeSi was also observed in runs SCO-6 and SCO-9. Fe_5Si_3 is known to decompose to FeSi and Fe_3Si above 18 GPa and 1300 K in the hydrogen free system[85]. However, this phase was observed in a H-bearing medium in a recent experiment[48]. Unlike ref.[48] where hydrogenation of the hexagonal Fe_5Si_3 phase in a H medium results in significant volume expansion, the unit-cell volume of the Fe_5Si_3 phase in our study is very close to that expected for H-free Fe_5Si_3 (ref.[86]), implying minimal hydrogen incorporation into this phase in our experiments.

FeH_x As in the other experiments. FeH_x is observed in the fcc structure after high-temperature heating and the dhcp structure before heating or away from the heating center. The diffraction intensity is weak compared to that of Fe_5Si_3 and the complex diffraction pattern (Extended Data Fig. 5) precludes the use of the accurate fitting and refinement of the unit-cell parameters of the FeH_x phases. However, the observed peak positions are consistent with those expected for $x \approx 1$ in FeH_x for both the dhcp and the fcc phases.

MgO MgO was observed throughout the runs in this pressure range, indicating breakdown of silicates upon melting in a hydrogen medium.

Ringwoodite/Stishovite Off from the hotspot center, weak peaks of ringwoodite were observed after heating (Extended Data Fig. 5). These diffraction peaks are much weaker than those of Fe_5Si_3 and MgO, suggesting a very small amount of silicate remains. Often at the center of heated spot, because of the melting of the sample and subsequent reaction, not much starting material remained but instead the area was filled with the hydrogen medium. Therefore, high-quality diffraction patterns are normally measured at spots away from the heating center after temperature quench. The temperature during heating at the spot where the diffraction patterns showing ringwoodite were measured was lower than the temperature at the heating center and was likely not high enough to achieve melting. The lack of melting may lead to the incomplete decomposition of the silicate by hydrogen, which is much more significant when silicate is molten. Stishovite was observed in SCO-7, 8, and 9. However, as shown in Extended Data Fig. 5, their diffraction intensities are low and therefore the amount in the samples is small.

4 SCO-10, 11, 12, 13a, and 13b

$Fe_{1-y}Si_y$ B2 $Fe_{1-y}Si_y$ was observed in runs SCO-10, 11, and 13b where temperature was sufficiently high to melt the silicate. The volume of B2 $Fe_{1-y}Si_y$ is close to the volumes reported for the same phase for Si/Fe = 1 (or $y = 0.5$) (ref.[76]) at high pressure and through decompression, suggesting little incorporation of H, consistent with the observations in ref.[77] that this phase cannot take in a large amount of H. At 1 bar, the unit-cell volume of B2 $Fe_{1-y}Si_y$ is 21.45±0.02 Å^3, slightly expanded from the value of 21.30 Å^3 for

$y = 0.5$ reported by ref.[76] (run SCO-13b). This is likely because there is a slight superabundance of Fe ($Fe_{1-y}Si_y$, $0.33 < y < 0.5$).

FeH_x We observed FeH_x in all the runs with a volume corresponding to $x \approx 1$. After pressure quench to 1 bar, FeH_x converts to bcc Fe (run SCO-13b). Bcc Fe has a unit-cell parameter of 2.8666±0.0001 Å^3 in agreement with the known value of pure Fe metal, 2.867 Å (ref.[83]), suggesting no Si incorporation in FeH_x at this pressure range.

Ferropericlase and bridgmanite At 1 bar after decompression of the heated samples, ferropericlase has a unit-cell volume of 74.70±0.02 Å^3, in agreement with the value of 74.71 Å^3 for endmember MgO (i.e., periclase)[87] (run SCO-13b). By contrast, the unit-cell volume of the remnant perovskite phase is 163.01±0.05Å^3, expanded from the unit-cell volume of endmember $MgSiO_3$ perovskite, 162.49Å^3 (ref.[88]) (run SCO-13a). The volume is marginally larger than the expected volume for $(Mg_{0.9}Fe_{0.1})SiO_3$ of 162.79 Å^3. Utilizing a linear interpolation between the unit-cell volumes for 10 and 20 mol% Fe from ref.[88] gives an approximate composition of $(Mg_{0.86}Fe_{0.14})SiO_3$. Note that the bridgmanite was observed in an area away from the center heated to lower temperatures below melting of the starting materials. When temperature was sufficiently high for melting of silicate (SCO-10, 11, and 13b), bridgmanite diffraction lines are either absent or weak, suggesting that most Si^{4+} was reduced to Si^0 or released as SiH_4.

5 Summary on the SIL and FAY runs

Pure SiO_2 mixed with Fe metal was also heated in a hydrogen medium. At 14 GPa after one heating event at 2899 K (SIL-2), SiO_2 partially breaks down to alloy with the mixed Fe and form B2 $Fe_{1-y}Si_y$ (Extended Data Fig. 6), consistent with Si^{4+} reduction. Si^{4+} in silica can also dissolve into dense hydrogen liquid as SiH_4 as shown in our experiments with fayalite below (Extended Data Fig. 4 and Supplementary Discussion 9) and ref.[26]. Some silica still remained after heating, and the observation is likely due to lower-temperature heating below melting. Raman measurements of the sample after laser heating at high pressure detected H_2O (Extended Data Fig. 3). Consistent results were obtained from other runs at 14–39 GPa (Supplementary Discussion 6).

When fayalite, Fe-endmember olivine (Fe_2SiO_4), was heated to 3017 K at 6 GPa, it broke down completely to form cubic metal phases (fcc, bcc, and B2) (Fig. 2b). The fcc phase is FeH_x with volumes indicating x as high as 2 to 2.3 (Extended Data Fig. 7; Supplementary Discussion 7). In this experiment without Mg, the silicate melt is completely consumed by reaction with H and the released O reacts with H to form H_2O, which is confirmed by Raman spectroscopy (Supplementary Discussion 7).

Above 10 GPa, fayalite breaks down to form silica and FeH_x (Supplementary Discussion 8 and Extended Data Fig. 8). No clear evidence for an Fe-Si phase was detected. The presence of H_2O is confirmed via Raman spectroscopy. The existence of silica diffraction lines after heating indicate that some Si remains oxidized at this higher pressure range. When fayalite (which has 10 times as much Fe^{2+} as San Carlos olivine) is used, much more H_2O can be rapidly produced, likely because it is much easier for Fe^{2+} to be reduced by H than Si^{4+} and Mg^{2+}. The rapid production of H_2O will raise the activity of H_2O and therefore suppress the reduction of Si^{4+}, which is more difficult than the reduction of Fe^{2+}. In runs where fayalite was melted with a much smaller amount of hydrogen (an Ar medium with 50% H_2; Supplementary Discussion 9), SiH_4 was detected in Raman measurements (Extended Data Fig. 4). Si remains oxidized in SiH_4, and therefore water production can continue under more oxidizing conditions through the formation

of SiH_4 (reaction 2).

6 SIL runs

FeH_x Although with a larger uncertainty due to the weak diffraction intensity of FeH_x, in (run SIL-1), the fcc phase showed a unit-cell volume of 50.7±0.2 Å^3 at 14 GPa after temperature quench, slightly lower than FeH_x with $x = 1$. Similar results were also seen in other SIL runs (Extended Data Fig. 6).

Coesite/Stishovite Because SiO_2 does not couple efficiently with the near infrared laser beam but is heated mainly through the coupling of Fe metal particles nearby, the heating was less efficient than in olivine, which contains Fe and directly couples with the laser beams. In these runs, temperature was not high enough to melt silica at this pressure (Extended Data Fig. 1). Therefore, some silica phases remained after heating. Some coesite peaks were identified in some runs (SIL-1 and SIL-2) despite the fact that the pressures were higher than the stable condition for coesite. Similar observation was also made in the FAY-3 and FAY-4 runs. This may indicate that hydrogen could affect the stable conditions for coesite.

$Fe_{1-y}Si_y$ In all SIL runs some Si^{4+} was reduced to Si^0 which alloyed with Fe present to form B2 $Fe_{1-y}Si_y$. Although due to the coupling considerations mentioned above along with the high melting temperature of silica, the amount (and thus diffraction intensity) was quite small. Raman modes of O-H bonding were detected around the heated area but not in unheated areas, suggesting that the SiH_4 formation (reaction 2) may play a major role for the H_2O formation.

7 FAY-1 and 2

$Fe_{1-y}Si_y$ A B2 phase with a unit-cell volume 7.2% larger than stoichiometric FeSi (or $y = 0.5$, ref.[76]) is observed (Extended Data Fig. 9). This is likely due to a slight excess of Fe (i.e. Fe/Si>1) which has a higher atomic radius than Si, rather than hydrogenation of FeSi which has not been observed in previous experiments with Fe-Si alloys under a H medium[77].

FeH_x In runs FAY-1 and FAY-2, the volume of the fcc metal is 23.6–27.5% larger than the volume of stoichiometric FeH reported at this pressure (∼51.3 Å^3) (ref.[80]) after heating. Following the method of ref.[74], we obtained $x = 2$–2.3 for the FeH_x (Extended Data Fig. 7). A very large volume expansion for fcc was also found for (Fe,Ni)H_x and FeH_x melted at pressures above 77 GPa (ref.[74]). In that case it was estimated that x was 1.8, slightly smaller but comparable to our observation. Upon decompression to 1 bar, the fcc phase disappears, as expected for FeH_x, which is known to lose hydrogen below ∼3.5 GPa (ref.[79]) (Extended Data Fig. 9).

Bcc phase A bcc phase with a unit-cell volume 1.7% larger than pure bcc Fe metal was observed. While bcc Fe metal is not observed in the pure Fe-H system at pressures above 3.5 GPa and temperatures this high[80], ref.[89] showed that a small amount of Si can stabilize bcc Fe beyond its standard stability field. It is likely our observation of the bcc phase is due to slight incorporation of Si. The volume expansion of 1.7% may be due to hydrogenation but is far smaller than the ∼20–25% volume expansion seen in FeH phases[79,80], consistent with previous observations that Si (which may be stabilizing this structure) inhibits the hydrogenation of Fe metal[77]. Upon decompression to 1 bar, the B2 and bcc phases remain stable.

8 FAY-3, 4, and 5

Above $\sim$18 GPa without H, fayalite is known to break down into FeO and stishovite[63]. We also observed the same trend in our experiments in the presence of hydrogen. However, in our hydrogen case, FeO reacts with hydrogen and forms FeH_x (Extended Data Fig. 8). Somewhat lower degree of Si loss was found in these runs. At this high pressure range, a much smaller volume of hydrogen can be heated conductively because of the thinning of the hydrogen medium, resulting in a larger axial thermal gradient in LHDAC. If this is the case, a smaller amount of liquid hydrogen may not be able to dilute H_2O enough to maintain a low H_2O activity in the heated area, reducing the extent of hydrogen-silicate reaction.

Silica Silica is present as both stishovite and coesite. When the molten sample is being temperature quenched, because of the positive Clapeyron slope of the coesite-to-stishovite transition, coesite may crystallize first at higher temperatures followed by crystallization of stishovite at lower temperatures[90]. Upon decompression to 1 bar, both coesite and stishovite remain. After decompression to 1 bar, stishovite was found to have slightly larger unit-cell volume ($V = 46.67 \pm 0.08$ Å^3) compared to 46.50 Å^3 reported by ref.[91] for anhydrous stishovite. H_2O can be incorporated in the crystal structure of stishovite and increase the unit-cell volume[92]. A previous study[92] reported the relationship between the unit-cell parameters and water content in hydrous stishovite. Utilizing that calibration, the change in unit-cell volumes implies $\sim$0.7 wt% H_2O in stishovite. However, the minimal change in the axial ratio relative to anhydrous stishovite ($c/a = 0.640$ compared to $c/a = 0.641$ reported by ref.[93]) does not align with an increase in the axial ratio reported in ref.[92] for hydrous stishovite. Therefore, the estimation of H_2O content should be interpreted with caution.

FeH_x At this pressure range, we observed $x = 0.8$–1 for fcc FeH_x. Upon decompression to 1 bar, fcc FeH_x reverts to bcc Fe metal. The unit-cell parameter of the bcc phase is 2.8677±0.0003 Å, which agrees with the expected value of pure Fe metal of 2.867 Å (ref.[83]), suggesting that all hydrogen has left the crystal structure of Fe metal upon decompression.

9 FAY-6, 7, and 8

Additional experiments on fayalite were conducted at similar conditions to runs FAY-1, 2, 3, and 4, but with a medium of a 1:1 ratio (by volume) of H:Ar instead of pure H_2. The solid phase products were the same as in the pure H-medium experiments. Not only does the mixed medium allow us to examine the reaction for low H_2 concentration, the setup also enhances the thermal and mechanical stability of the medium, particularly during decompression to obtain Raman spectra of SiH_4 (Extended Data Fig. 4).

10 Estimation for the pressure-temperature conditions of the atmosphere-interior boundaries of super-Earths and sub-Neptunes

In order to define the planet population in which H_2O production takes place we examined in which planets the silicate-hydrogen boundary meet the experimental pressure-temperature range along their thermal evolution.

We run interior evolution models of planets with rocky core and H,He envelopes, based on ref.[28], varying

the planet mass, envelope mass, and planet initial thermal state (derived from planet formation assumptions). The estimation shows that rocky super-Earth to sub-Neptune planets, in the mass range of 3–15 M_E with 2–20 wt% of gas (H, He) are expected to experience H_2O production. In planets with $M_p < 3 M_E$ and/or $<2\%$ H_2, the core-envelope boundary (CEB) is below the experimental pressure range, while in planets with $M_p > 15 M_E$ and/or $>20\%$ H_2 temperature is above the experimental range. The planet mass and envelope mass ranges found here are an order of magnitude estimate, and specific planet formation or structure evolution conditions can vary these values slightly.

Based on the model results, we constructed structure-evolution profiles of super-Earth and sub-Neptune planets under various conditions. In Fig. 2, we show an example of such structure-evolution profile for a $5 M_E$ super-Earth planet with 5% H + He envelope. Shown is the temperature (color) profile in the interior from center to 1 bar pressure, as a function of time. The experimental pressure-temperature range is shown in black (dotted and solid curves, respectively) and the CEB is in dashed red. As can be seen, the CEB of this planet stays in water production regime for many giga years. Importantly, heat transport in most of the interior is found to be by large scale convection along the evolution track.

11 Estimated quantities of reactants and products in the LHDAC experiments

The reaction observed in our experiments on olivine melt with Fe metal in a hydrogen medium can be described as the following:

$$\begin{aligned}&(Mg_{0.9}Fe_{0.1})_2SiO_4 + (a-0.2)Fe + [2(2-z)+0.5x(a-(z/y))]H_2\\&\rightarrow 2MgO + (z/y)FeSi_y + (a-(z/y))FeH_x + (1-z)SiH_4 + 2H_2O,\end{aligned} \quad \text{(Extended Data Reaction 1)}$$

where a is the total amount of Fe in the system including Fe metal loaded together with olivine and Fe^{2+} in olivine, which is 0.86. y and x are the amounts of Si and H in $Fe_{1-y}Si_y$ and FeH_x after reaction, respectively. They can be obtained from the measured unit-cell volumes combined with composition-volume relations[74,76]. z is the fraction of Si reduced to metal and can be estimated by comparing diffraction intensities and amount of Si in Fe-Si alloy, i.e., y.

In an LHDAC experiment, amount of heated medium, H_2 in our case, is difficult to measure. However, some observational constraints can be used for an estimation. The volume of warm dense liquid hydrogen involved in the reaction should be limited to that in the grain boundaries of the heated part of the olivine + Fe metal sample foil and the medium layers adjacent to the heated sample surfaces along the loading axis of DAC (Fig. 1a). It should be limited laterally as well, to the laser beamsize, because of the radial thermal gradients in LHDAC. From these constraints, we found that 4.5–5.7 wt% H_2 existed in the heated volume together with ~76 wt% silicate and ~19 wt% Fe metal in our sample setup before the reaction.

The amount of H_2O produced from the reaction can be much better estimated. First, concerning the Si effect only, both Fe-Si alloy formation and SiH_4 formation produce the same amount of H_2O per olivine as these reactions release the same amount of O per olivine from molten silicate to liquid hydrogen. Second, in our olivine + Fe metal runs, all Si in silicate was released at lower pressures (6–8 GPa) and almost entirely at higher pressures (>25 GPa). Therefore, the involved parameters, such as x, y, and z, in reaction Extended Data Reaction 1 do not affect the estimation for the amount of total H_2O produced, but it depends only on the amount of melted olivine: 2 moles of H_2O can be produced from 1 mole of molten

olivine. According to our estimation, 18.1(5) wt% H_2O was produced by Extended Data Reaction 1. This calculation is provided in Supplementary Code 1.

We also provide Supplementary Code 2 for the case of partial reaction and Supplementary Code 3 for the effects of Mg/Si ratio.

12 Segregation of iron alloys in a magma ocean

Once the liquid Fe metal alloy is fully sequestered from the silicate melt by density to form a stratified metallic layer below the silicate melt layer, the reduction of Si may stop. However, the formation of SiH_4 can continuously liberate oxygen from silicates to form water without the need to reduce Si^{4+}. On the other hand, liquid droplets of Fe metal alloy—if sufficiently small in size—could be suspended in silicate melt, delaying metal-rock segregation in the molten interior of sub-Neptunes[94,95], an effect that could be further intensified by the decrease in metal droplet density due to alloying of Fe with H and Si present in the magma ocean. In this case, the Fe-Si alloy formation may continue to contribute to the endogenic production of water.

13 Endogenic water production contributed by magnesium involved reactions

In ref.[23] and this study, MgO remained after the hydrogen-magma reaction, mainly because the temperatures were lower than the melting of the MgO component. In more recent experiments where MgO and Fe metal were melted together in a hydrogen medium, it was found that the Mg–O bond breaks down to form Mg hydrides[24]. The reaction also results in the release of O and formation of water, similar to the case of SiH_4 formation in this study. However, because much less Mg hydride was produced in that reaction, its contribution to the total amount of endogenic water should be much smaller than that from Si release. On the other hand, because Mg hydride formation occurs at higher temperatures, endogenic water production can occur from at least $\sim$5000 K.

The study[24] also observed crystallization of Mg_2FeH_6 after heating at pressures below 13 GPa. Although the melting temperature of Mg_2FeH_6 is not known to our knowledge, from the melting temperatures of related materials, such as MgH_2 and FeH (refs[96,97]) at the pressure range, it should be lower than 1500 K if the material undergoes incongruent melting. Therefore, Mg_2FeH_6 is unlikely to impact the hydrogen-magma reaction we report here until a sub-Neptune cools down to sufficiently low temperatures to crystallize the phase. On the other hand, the hydride bond formation will lower the melting temperature of the magma and extend the molten state of the interior and facilitating more efficient mixing and chemical reacting between the hydrogen and magma.

14 Impact of SiH_4 and MgH_2 on the dynamics of the interior

Although, to our knowledge, no direct experimental measurements or computational simulations have been performed, experiments have shown that SiH_4 and H_2 are miscible in the fluid state up to 6 GPa and 300 K (ref.[98]). Furthermore, SiH_4 and H_2 can form a solid solution (i.e., miscibility in the solid state) at

high pressures[98,99]. Therefore, under the P–T conditions relevant to the CEB of sub-Neptunes, SiH_4 and H_2 are expected to exist as a single fluid.

To our knowledge, the density of fluid SiH_4 is not known at the P–T conditions of the CEB. However, useful insights can be gained by comparing the solid phases of H_2O and SiH_4, as their equations of state have been measured at high pressures and 300 K (refs[100,101]). We find that the density of SiH_4 is 20–30% lower than that of H_2O over the pressure range considered in this study. Similarly, the density of MgH_2 is slightly lower than that of H_2O (refs[102,103]). This is primarily due to the significantly larger molar volumes of SiH_4 and MgH_2 compared with H_2O.

There are several explanations for the larger molar volumes of these hydrides relative to H_2O. The most notable is the difference in the oxidation state of hydrogen: in SiH_4 and MgH_2, hydrogen exists as H^-, whereas in H_2O, it is H^+. Because H^- has two electrons and only one positive nuclear charge, it has a much larger volume (ionic radius of 1.34 Å) than H^+ (ionic radius of 0.18 Å), resulting in significantly larger volumes for SiH_4 and MgH_2 compared to H_2O.

The larger molar volumes of MgH_2 and SiH_4 result in lower densities relative to H_2O under the relevant P–T conditions. As a result, a mixture containing MgH_2, SiH_4, and H_2O has a lower density than pure H_2O. In planetary interiors, large-scale convection is an efficient mechanism for heat and material transport. This process is driven by buoyancy forces arising from density gradients due to thermal or compositional differences[104]. Since the strength of convective-mixing is inversely related to the density variation between the fluid and its surroundings (e.g., Appendix in ref.[56]), the reduced density of the mixture enhances the efficiency of convective-mixing and prolongs its activity.

If, under certain conditions the MgH_2, SiH_4, and H_2O mixture would result in higher density than that of a pure water that we considered in our simulations, a semi-convection (double diffusive convection[105]) may develop instead of large-scale convection. Semi-convection would limit the mixing efficiency and consequently the water production may stop earlier. However, these conditions were not found in our examination.

References

75. Ji, C. *et al.* Ultrahigh-pressure isostructural electronic transitions in hydrogen. *Nature* **573**, 558–562 (2019).

76. Fischer, R. A. *et al.* Equations of state in the Fe-FeSi system at high pressures and temperatures. *J. Geophys. Res. Solid Earth* **119**, 2810–2827 (2014).

77. Fu, S., Chariton, S., Prakapenka, V. B., Chizmeshya, A. & Shim, S.-H. Hydrogen solubility in FeSi alloy phases at high pressures and temperatures. *Am. Minerol.* **in press**, DOI: https://doi.org/10.2138/am-2022-8295 (2022).

78. Slater, J. C. Atomic radii in crystals. *The J. Chem. Phys.* **41**, 3199–3204 (1964).

79. Badding, J., Hemley, R. & Mao, H. High-pressure chemistry of hydrogen in metals: In situ study of iron hydride. *Science* **253**, 421–424 (1991).

80. Narygina, O. *et al.* X-ray diffraction and mössbauer spectroscopy study of fcc iron hydride FeH at high pressures and implications for the composition of the Earth's core. *Earth Planet. Sci. Lett.* **307**, 409–414 (2011).

81. Hirao, N., Kondo, T., Ohtani, E., Takemura, K. & Kikegawa, T. Compression of iron hydride to 80 GPa and hydrogen in the Earth's inner core. *Geophys. Res. Lett.* **31** (2004).

82. Jain, A. *et al.* The Materials Project: A materials genome approach to accelerating materials innovation. *APL Mater.* **1**, 011002, DOI: 10.1063/1.4812323 (2013).

83. Rotter, C. A. & Smith, C. S. Ultrasonic equation of state of iron: I. low pressure, room temperature. *J. Phys. Chem. Solids* **27**, 197 (1966).

84. Utsumi, W., Weidner, D. J. & Liebermann, R. C. Volume measurement of MgO at high pressures and high temperatures. *Geophys. Monogr. Geophys. Union* **101**, 327–334 (1998).

85. McGuire, C., Santamaria-Pérez, D., Makhluf, A. & Kavner, A. Isothermal equation of state and phase stability of Fe_5Si_3 up to 96 GPa and 3000 K. *J. Geophys. Res. Solid Earth* **122**, 4328–4335 (2017).

86. Errandonea, D. *et al.* Structural stability of Fe_5Si_3 and Ni_2Si studied by high-pressure x-ray diffraction and ab initio total-energy calculations. *Phys. Rev. B* **77**, 094113 (2008).

87. Fei, Y. & Mao, H.-k. In situ determination of the nias phase of FeO at high pressure and temperature. *Science* **266**, 1678–1680 (1994).

88. Mao, H. *et al.* Effect of pressure, temperature, and composition on lattice parameters and density of $(Fe,Mg)SiO_3$-perovskites to 30 GPa. *J. Geophys. Res. Solid Earth* **96**, 8069–8079 (1991).

89. Lin, J.-F., Heinz, D. L., Campbell, A. J., Devine, J. M. & Shen, G. Iron-silicon alloy in Earth's core? *Science* **295**, 313–315 (2002).

90. Akimoto, S., Yagi, T. & Inoue, K. High temperature-pressure phase boundaries in silicate systems using in situ x-ray diffraction. In *High-Pressure Research*, 585–602 (Elsevier, 1977).

91. Sinclair, W. & Ringwood, A. Single crystal analysis of the structure of stishovite. *Nature* **272**, 714–715 (1978).

92. Nisr, C. *et al.* Large H_2O solubility in dense silica and its implications for the interiors of water-rich planets. *Proc. Natl. Acad. Sci.* **117**, 9747–9754 (2020).

93. Andrault, D., Fiquet, G., Guyot, F. & Hanfland, M. Pressure-induced landau-type transition in stishovite. *Science* **282**, 720–724 (1998).

94. Lichtenberg, T. Redox hysteresis of super-earth exoplanets from magma ocean circulation. *The Astrophys. J. Lett.* **914**, L4 (2021).

95. Young, E. D., Stixrude, L., Rogers, J. G., Schlichting, H. E. & Marcum, S. P. Phase equilibria of sub-Neptunes and super-Earths (2024). 2408.11321.

96. Moser, D. *et al.* The pressure–temperature phase diagram of MgH_2 and isotopic substitution. *J. Physics: Condens. Matter* **23**, 305403, DOI: 10.1088/0953-8984/23/30/305403 (2011).

97. Fukai, Y., Mori, K. & Shinomiya, H. The phase diagram and superabundant vacancy formation in Fe–H alloys under high hydrogen pressures. *J. Alloy. Compd.* **348**, 105–109, DOI: 10.1016/S0925-8388(02)00806-X (2003).

98. Wang, S., Mao, H.-k., Chen, X.-J. & Mao, W. L. High pressure chemistry in the H_2-SiH_4 system. *Proc. Natl. Acad. Sci.* **106**, 14763–14767, DOI: 10.1073/pnas.0907729106 (2009).

99. Strobel, T. A., Somayazulu, M. & Hemley, R. J. Novel pressure-induced interactions in silane-hydrogen. *Phys. Rev. Lett.* **103**, 065701, DOI: 10.1103/PhysRevLett.103.065701 (2009).

100. Sugimura, E. *et al.* Compression of H_2O ice to 126 GPa and implications for hydrogen-bond symmetrization: Synchrotron x-ray diffraction measurements and density-functional calculations. *Phys. Rev. B* **77**, 214103, DOI: 10.1103/PhysRevB.77.214103 (2008).

101. Degtyareva, O. *et al.* Crystal structure of SiH_4 at high pressure. *Phys. Rev. B* **76**, 064123, DOI: 10.1103/PhysRevB.76.064123 (2007).

102. Moriwaki, T., Akahama, Y., Kawamura, H., Nakano, S. & Takemura, K. Structural phase transition of rutile-type MgH_2 at high pressures. *J. Phys. Soc. Jpn.* **75**, 074603, DOI: 10.1143/JPSJ.75.074603 (2006).

103. Cui, S., Feng, W., Hu, H., Feng, Z. & Wang, Y. Structural phase transitions in MgH_2 under high pressure. *Solid State Commun.* **148**, 403–405, DOI: 10.1016/j.ssc.2008.09.033 (2008).

104. Kippenhahn, R. & Weigert, A. *Stellar Structure and Evolution*. Astronomy and Astrophysics Library (Springer-Verl, Berlin Heidelberg Paris [etc.], 1991).

105. Turner, J. S. & Stommel, H. A New Case of Convection in the Presence of Combined Vertical Salinity and Temperature Gradients. *Proc. Natl. Acad. Sci.* **52**, 49–53, DOI: 10.1073/pnas.52.1.49 (1964).
